# On the Medicinal Powers of Elaterium

**Published:** 1823-06

**Authors:** 


### Art. VII.—*On the Medicinal Powers of Elaterium.* By Dr. Kinglake.

The medicinal powers of elaterium have been always regarded as being peculiarly and violently active; so much so, indeed, as to have precluded them from free and confident use. There are but few articles in the materia medica that exert a stronger influence on the actions of life than elaterium; and experience has ascertained that this agency may, in various conditions of disease, be turned to a highly salutary account. Substances may possess qualities capable of deeply affecting the actions of animal life, without at all proving beneficial. When this is the case, the agent is destitute of medicinal or remedial powers. Adequate experience can alone determine the real efficacy of reputed medicines; and to estimate correctly, by even this source of intelligence, the closest and most cautious observation is required. The early periods of medical history have recorded the medicinal virtue of elaterium, and it is probable that, if its active properties had not occasionally operated with a violence that induced an apprehension of its being unsafe for general use, it would long since have acquired an established reputation for being almost singularly efficacious in speedily and permanently removing serous effusions, constituting the various forms of dropsical affection.

The Greeks gave to the juice of the wild cucumber *(cucumis sylvestris,)* the name of *elaterion*, whence is derived elaterium, denoting a strong purgative quality. Theophrastus, who flourished three hundred years before the Christian era, regarded it as a medicine of great and various power, affirming that it would retain its active properties two hundred years; and that, when fifty years old, it would, by its evaporable force, extinguish a lighted candle or lamp, when nearly approached to it. Pliny also observes, that a test of the genuineness and purity of elaterium, is when "it causes a lighted candle to sparkle upwards and downwards." The medicinal virtues of elaterium were not unknown to Hippocrates and Galen: they speak of them as being violently purgative. Later observers have investigated the qualities of elaterium, and have ascertained them to possess higher and more efficient cathartic powers than those of any other article of the materia medica. The German Zuingerus, in his Theatrum Botanicum, remarks, "Diese Gurke hat auch eine etzende purgirende Salz bey sich, vermittelat dessen es nicht nur von sich den Leib purgient sondern auch den monatsfluss der Weibern befördent, dieser Saft halt sich sehr viele Jahren, und verlient seine Kraft nieht."

The constituent principles of elaterium possess qualities that specifically affect the peristaltic power of the intestinal canal.

They not only excite it to increased action, but seem also vastly to augment the whole of the enteric secretions; so that the alvine discharges resulting from the operation far exceed in quantity those which are produced by any other known purgative. The afflux of fluids derived to the intestines by this excitement, and the consequent effusion into their cavity, largely abstract from the superfluous water of the circulating fluids, and thus room is afforded for absorption from the visceral and cellular cavities in dropsical disease. Such is the strong influence which this medicine exerts on the vital power of the first passages, that doses from a quarter of a grain to one grain, taken night and morning, will induce and sustain a cathartic action, that will remove from the system, through the intestines, from two to four quarts of fluid in the course of twenty-four hours.

This vast excretion creates a deficiency of fluids in the sanguiferous system, and thereby causes the absorbents freely to receive from the cavities any serous accumulation that may have taken place in them. If this absorbing and evacuating operation be sufficiently carried on, all dropsical distention, and the various hurtful oppressions connected with such plenitude, will be removed, and an opportunity afforded, in the absence of visceral disease, for a permanent re-establishment of the healthful tone and energy of life.

Elaterium does not appear to produce its full effect as an hydragogue until after it has been taken for a week or ten days, when its specific or peculiar influence on the intestines seems to be established, and to proceed uninterruptedly as long as it may be judged right to persist in its use. The stomach is occasionally much nauseated by elaterium; but this early effect of the remedy speedily passes on to the bowels, where its active influence at once relieves the stomachic disturbance, and carries off, by copious discharges from the intestinal canal, large quantities of effused and oppressive fluids. Nor does the sickening effect of the medicine impede its salutary action in promoting the absorption and discharge of serous accumulations. Like digitalis, it is probable that, by temporarily diminishing and retarding the contractile energy of the arterial system, it lessens exhalation, whilst absorption proceeds in an increased proportion. In dropsical disease, it frequently happens, in addition to the general atony prevailing in these cases, that a morbid determination obtains in the exhalent division of the arterial system, which occasions an inordinate supply of fluid in all the interstitial spaces of the body. This occurs without proportionate absorption, and, of course, must speedily deluge both the cellular and visceral cavities, into which it is liable to be diffused. Suspending and diverting this determination by the nauseating influence of medicine like elaterium, exerting its concentrated action on the peristaltic power of the intestines, may efficiently remove both the cause and effect of the redundant exhalation.

It is not evident, when elaterium is operating most powerfully in abstracting from the bowels the aqueous accumulations existing in dropsical disease, that it augments the urinary secretion. Indeed, during its full influence on the intestines, it is hardly possible that its action could be also extended to the kidneys. Its cathartic effect is so direct and intense as almost necessarily to confine its undivided power to that sphere of action. Were it more distributed, its evacuant agency would be proportionately diminished. When cathartic substances strongly excite the peristaltic power of the intestines without producing much evacuation, the excitement is apt to be sympathetically transferred to the kidneys, and to be shown in an augmented secretion of urine. Thus cathartic medicines have been held to be likewise diuretics, when exhibited in suitable quantities. This notion is, perhaps, more imaginary than true; and, were it admissible, it would suppose a convertible property in all medicines, in denial of the prevailing and more reasonable opinion, that medicinal substances, in the vast diversity of their component parts, have properties peculiarly adapted to produce specific effects on the different states and conditions of excitability obtaining in the various and dissimilar textures of the human body. The natural aptitudes subsisting between the peculiar excitability of different parts of the system and the stimulant properties of certain medicinal substances, must be accurately investigated and known before the desired precision can be obtained in determining the specific powers and uses of such agents.

A medicine so active as elaterium may probably be employed in doses so small as to be incapable of affecting the first passages, as an useful alterative, in chronic diseases, originating as well from vitiated excitement in structures not disorganized, as in morbid enlargement from change of texture and growth of new parts. The whole class of glandular diseases, whether occurring in visceral organs or in glands themselves in their insulated situations, presents the sort of distempered inveteracy that would require all the subtilty and power appertaining to elaterium to overcome it. Dr. Ferriar, in his Medical Reports, has suggested that this medicine might prove an efficacious alterative in obstinate diseases of long standing. Impressed with a similar persuasion, it has been already subjected to trial under my direction;—but, it must be confessed, not sufficiently even to warrant a report of its effects, much less to decide on what it may, under a more extended and varied application of its powers, be capable of accomplishing. The form in which it has been ordered by me, has been a solution of one grain of the extract of elaterium in one ounce of proof spirit; of which thirty drops have been administered as a dose, three times a-day. This dose, if the solution of the medicine be accurate, will be equivalent to a sixteenth-part of a grain of the extract in substance; and even this small portion has been known to nauseate. For children, it would be advisable to begin with ten drops, and to advance the number gradually until sickness may be induced, which should be regarded as a test of the maximum dose. By slow and progressive augmentation, it may be ultimately borne to the extent of an eighth, or even a quarter of a grain, without disordering either the stomach or bowels. But the ability to take the increased dose arises rather from a familiarity obtaining between the excitability of the stomach and the medicine, than from an inefficiency of the latter. The influence of elaterium will proceed to a final point of saturation, when it will be no longer borne. To attempt to urge it beyond that extent, would be to overpower vital action in a manner that would be distressing to the patient's feelings, and may even endanger life itself.

After powerful medicines, such as elaterium, mercury, arsenic, zinc, antimony, prussic acid, opium, digitalis, colchicum, the various narcotics, &c. have been taken long enough to disorder either the stomach or head, they should be discontinued for an interval sufficiently long to allow such saturated or redundant influence to subside, when they may be respectively resumed at the lowest doses, and gradually carried as far as the curative indication may appear to require.

*Taunton; March 9th,* 1823.

